# Delayed tracking and inequality of opportunity: Gene-environment interactions in educational attainment

**DOI:** 10.1038/s41539-022-00122-1

**Published:** 2022-05-04

**Authors:** Antonie Knigge, Ineke Maas, Kim Stienstra, Eveline L. de Zeeuw, Dorret I. Boomsma

**Affiliations:** 1 Department of Sociology/ICS, Utrecht University, Utrecht, The Netherlands; 2 Department of Sociology, Vrije Universiteit Amsterdam, Amsterdam, The Netherlands; 3 Department of Biological Psychology, Vrije Universiteit Amsterdam, Amsterdam, The Netherlands

**Keywords:** Education, Human behaviour, Sociology, Policy, Education

## Abstract

There are concerns that ability tracking at a young age increases unequal opportunities for children of different socioeconomic background to develop their potential. To disentangle family influence and potential ability, we applied moderation models to twin data on secondary educational track level from the Netherlands Twin Register (*N* = 8847). Delaying tracking to a later age is associated with a lower shared environmental influence and a larger genetic influence on track level in adolescence. This is in line with the idea that delaying tracking improves equality of opportunity. Our results further suggest that this is mostly because delaying tracking reduces the indirect influence of family background on track level via the test performance of students. Importantly, delaying tracking improves the realization of genetic potential especially among students with low test scores, while it lowers shared environmental influence on track level for students of all test performance levels.

## Introduction

Most people agree that children from all walks of life should be able to reach their full potential. This is precisely the argument why all educational systems at some point sort students into different schools or classes based on their ability (varying from as early as age 10 in Germany to age 16 in, e.g., the US and the UK)^1,2^. Such between- or within-school ability tracks would make it easier to adjust the learning environment to the needs of students of diverse potential as opposed to differentiating the curriculum within the same classroom^3^. This presupposes that students are always sorted into the track that fits their potential ability, while there are concerns that sorting is also based on family background, especially when the tracking decisions are taken at a relatively young age. The weight of empirical evidence shows indeed that the influence of family background on educational attainment is stronger in educational systems where tracking occurs at a younger age, while average performance is not higher (for reviews of the literature, see refs. ^2,4,5^). These studies interpret this as evidence that tracking at a young age does not allow everybody to make most of their talents.

However, the way in which studies on inequality of educational opportunity typically test whether delaying tracking to a later age makes educational attainment depend less on family background and more on potential ability is to use parental socioeconomic status (SES) measures to capture family background and measures such as IQ to capture potential ability. This is problematic for several reasons. One issue is that it is virtually impossible to have measures for all the possible ways in which family background impacts educational attainment. In an attempt to get around this problem, researchers have used sibling similarity in educational attainment as an omnibus measure of family background^6^. But sibling similarity does not only reflect similarity in educational outcomes due to siblings sharing their family background, but also due to their genetic sharing. Sibling similarity thus mixes up the influences of family background and potential ability. Including measures of actual ability to capture potential ability is not a satisfying solution either. Similar to family background, it is difficult, if not impossible, to have measures for all the cognitive (e.g., verbal, information processing speed, memory) and non-cognitive (e.g., self-control, motivation, grit) abilities that are important in school^7,8^. Moreover, family influence starts at a very early age, possibly prenatally, so every test administered to capture ability will also reflect partly the influence of family background^9^.

This study makes use of a ‘genetics toolbox’ to address much of these problems. We analyze data from mono- and dizygotic twins to disentangle genetic influences from environmental influences shared by children from the same family and environmental influences specific to the individual. This approach is often referred to as the classical twin design, which allows for the estimation of heritability and the moderation of heritability and environmental impact by a measured variable^10,11^. It has been argued that heritability, or the variance explained by genetic influences, is a good overall measure for the opportunities that people have to realize their potential and shared environmental influence a good overall measure for the impact of family background^12^. If tracking at a later age increases the importance of potential ability and reduces the importance of family background, one would thus expect genetic influences to be larger and shared environmental influences to be lower in educational systems in which tracking occurs later.

Note that this hypothesis comes with caveats. First, genetic influence may not only reflect realization of "positive" potential but also of "negative" potential that can hamper education (think, e.g., of disorders), although positive traits seem to carry most weight^13,14^. Second, because the shared environment captures all environmental influences that make siblings more similar, it should be seen as a broad indicator of all the different ways in which family background matters, including parental resources and behaviors, neighborhood characteristics, and so on. On the one hand, this is a strength because it circumvents the impossible task of having to measure them all. On the other hand, downsides are that the shared environment forms a black box, and that it may capture things one does not want to. For example, without controlling for year of birth, the shared environment would reflect any differences in average educational attainment between age cohorts (for an excellent discussion, see ref. ^15^).

Although it is important to keep these issues in mind, others have suggested in a similar fashion that genetic influence should be larger and shared environmental influence lower in educational systems that promote equality of opportunity (e.g., see refs. ^16–20^). Empirical testing of this hypothesis is scarce and it has not been applied to tracking age practices. The first question we ask is therefore: how does the influence of genes and the shared environment on educational attainment depend on the age at which children are tracked? We define educational attainment as the level of secondary education, which is a strong determinant of final educational attainment.

If tracking at a young age generates inequality of opportunity, it is important to understand why this is the case. A key distinction in the literature is that between what has been labeled primary and secondary effects of family background. Family background matters for level of education first through impacting how children perform (in terms of test scores etcetera) (*primary effects*). On top of these performance differences, so even if children with different backgrounds perform equally, their levels of education tend to diverge because of different educational decisions (*secondary effects*). In our analyses, we separate how tracking age impacts these primary and secondary effects by distinguishing genetic and shared environmental effects related to performance from those net of performance.

The first reason why early tracking could amplify secondary effects is that it may increase the risk for children with a disadvantageous background to attain lower levels of education than would be expected based on their potential. We argue in this paper that if such *hampering* by disadvantage is the driving mechanism, a lower tracking age decreases genetic and increases shared environmental influence especially among high-performing students. Second, early tracking could introduce possibilities for advantaged children to attain higher levels than their potential merits. We argue that such *compensation* by advantage would lead to the opposite prediction: a lower tracking age decreases genetic and increases shared environmental influence especially among low-performing students. Because it is unclear if one of these two types of mechanisms is underlying secondary forms of inequality of opportunity, the second question we ask is: how does the moderation of genetic and shared environmental influence on educational attainment by tracking age depend on the performance level of children?

To answer these questions, we analyze data on *N* = 8847 twins from the Netherlands Twin Register. The Netherlands provides an excellent case to study the consequences of tracking. The tracking procedure starts at a relatively young age (at the end of primary school around age 12) into five tracks (the three lowest tracks prepare for senior secondary vocational training, a middle stream prepares for tertiary vocational college, and an academically oriented track gives access to university). Secondary schools, however, differ in how definite this tracking is. Some schools put students into one particular track immediately at the start of secondary school based on the track recommendation of the primary school teacher. Other schools do not decide immediately on the definitive track level of a student but first put students in a class that combines two or more levels. After observing students 1–3 years (depending on the school) in such a heterogeneous class, the secondary school teachers decide what track level is appropriate for a student. This variation allows us to study if delaying definite tracking to a later age has consequences for inequality of opportunity.

An advantage of studying tracking within one country is that other aspects of the educational system and country are held constant. It is not necessarily the case, however, that whether children are tracked immediately or later is completely exogenously determined. This means that the association of interest, i.e., between delaying tracking and genetic and environmental influences on educational attainment, could be (partly) spurious or suppressed. Dutch children, together with their parents, choose a secondary school from the pool that offers their track level. There are virtually no restrictions on choice (no catchment areas, low or no tuition, often many schools close by). Research shows that important criteria are distance to school, school quality, student composition, reputation, peer effects, pedagogical approach, and denomination, and some find that the weight of the criteria depends on parental SES and child’s performance level^21–23^. We are not aware of a study that explicitly examined how important a homogeneous versus a heterogeneous class is as a criterium. Although not the same, Herweijer and Vogels^24^ get somewhat close by asking how important parents find the number of tracks a school offers. They show that this criterium is relatively unimportant and unrelated to parental SES, denomination, political attitudes, and whether children go to a heterogeneous class or not. Although this is somewhat reassuring, attending a homogeneous versus heterogeneous class could still be correlated with other school characteristics that lead to selectivity. Van Elk et al. ^25^ report for those with a MAVO track recommendation (old label for VMBO-t) that children from higher-SES and more urbanized areas more often attend a heterogeneous class, but that none of the effects are significant in a multivariate analysis. Borghans et al. ^26^ show for those with a HAVO to VWO recommendation that higher-SES and higher-performing children are less likely to attend a heterogeneous class. All in all, the evidence does not point towards strong selectivity, but nevertheless we check whether two potential confounders, namely family SES and school performance of the child, influence the choice to delay tracking or not.

In the remainder of the introduction, we first provide a short theoretical background on how genes and the environment are thought to influence child development, and then we theorize how the tracking age of an educational system impacts these genetic and environmental influences.

In modern industrialized societies, heritability of cognitive ability as assessed by psychometric IQ tests, is estimated to be approximately 55% around the age of 12, i.e., 55% of the variation in cognitive ability is due to genetic differences between these children^27^. High heritability does not mean that cognitive development or other forms of development are coded in the genes and just emerge with maturation, leaving little influence of the environment^28^. Genetic differences between people only come to expression through people’s interaction with their direct environment^29^. To give an example: a child may have an aptitude for reading, but if there are no books around, this genetic potential will be left unrealized. Higher levels of heritability can thus be expected if the opportunities in the direct environment of children to develop their potential improve.

Transactional models argue that children select and are selected into different environments partly based on their genetic predispositions, and these environments in turn have an impact on their development^28,30,31^ In case of our reading example, it would mean that children with an aptitude for reading are more likely to pick up a book than children without this aptitude (active gene–environment correlation), and that parents are more likely to buy books for children with an eagerness to read than for children with little appetite for reading (evocative gene–environment correlation). This in turn means that the reading ability of children with a genetic predisposition for reading is stimulated more than that of children with a genetic makeup geared less towards reading. Based on this, it is expected that if the wider environment is such that children have autonomy in shaping their direct environment, genetic differences are allowed to be expressed in terms of developmental differences. Herd et al. ^32^ show, for example, that the genetic influence on educational attainment increased for women to become more like men’s during the period in which women’s access to education became less restricted in society.

An important societal context that impacts whether children realize their potential or not is the way the educational system is organized. Tracking children into different ability groups could in principle be a way to optimize children’s direct learning environment to their genetic predispositions. However, in practice, ending up in a certain track may not only be the result of genetic predispositions, but also in part of socioeconomic background^33^. In the exposition below, we hypothesize that the younger the tracking age, the less educational attainment is a result of children realizing their genetic potential and the more it succumbs to primary and secondary effects of family background.

Before doing so, it is good to clarify three things about the relation between genes and family background. First, while Jackson^34^ stresses that primary effects also include transmission of genetic material from parents to children, Boudon^35^ originally stressed only sociocultural factors. Parental SES can have a causal effect on children’s performance through these sociocultural factors, but not through the transmission of genes. Parental genes may causally influence parental SES (but not the other way around) and children’s performance (via children’s genes), which would create a spurious association between parental SES and children’s performance. When talking about primary (or secondary) *effects of* socioeconomic background, it thus makes sense to exclude genetic transmission mechanisms. Second, and related to the first, because parental genes may influence both parental SES and children’s genes, genes and family background can correlate (labeled passive gene-environment correlation: e.g., parents with an aptitude for reading do not only pass on these genes to their children but are also more likely to have books in the household)^36^. The presence of gene–shared environment correlation makes it more difficult to disentangle genetic and shared environmental influences, but in the methods section we discuss why we think that it is not very problematic in our case (see "Assumptions of the fitted twin models"). Third, based on theoretical ideas related to the ones we apply, it has been argued that children have more opportunities in high- than low-SES contexts to select and evoke their environment based on their genetic proclivities^31^. Genetic influences on cognition indeed have been found to be larger for children from high SES families in the US, but not in the Netherlands and other Western European countries^37^. For our measure of educational performance, heritability does not differ across parental SES either^38^. The potentially complicating matter of interaction between genes and parental SES is thus unlikely to play a role in the context of our study.

In the Netherlands, the tracking procedure at the end of primary school is based partly on a national standardized test score (CITO) and partly on teacher’s track recommendations^39^. There is a strong association between CITO-scores and socioeconomic background^40^. Moreover, De Zeeuw et al. ^38^ show that children with a low genetic propensity for educational attainment still tend to score high on the CITO-test if they are from high-SES background. This supports the idea that performance differences by family background do not only reflect differences in talent, but also the extent to which the home environment is conducive to children reaching their potential^34^. For example, compared to low-SES parents, high-SES parents have more economic resources to invest in e.g. private tuition and learning materials, and can transmit more cultural capital to their children, which helps them to do well in school^41^. The younger children are, the more their direct environment is shaped by their parents. When children grow up, they "gain increasingly more autonomy in selecting their peer groups, afterschool activities, academic courses, and other positive learning experiences" (Tucker-Drob et al., 2013, p. 351)^31^. This should enable these choices to be steered to a greater extent by genetic predispositions. In line with this, it has been shown that variance in cognitive ability due to the shared environment decreases from around 60% in infancy to virtually none in adolescence, while genetic variance increases from <25% to ~70%^31,42^ (if children’s own choices are not genetically steered, more autonomy would mean that shared environmental effects give way to non-shared environmental instead of genetic effects). A similar pattern could pertain to educational performance, which has considerable (genetic and shared environmental) overlap with cognitive ability in the Netherlands^43^. This would imply that, the younger the age at which performance differences are used to sort children into different educational levels, the less educational attainment will depend on genes and the more on the shared environment (i.e., *primary effects* become stronger). Primary effects do not only depend on the influence of family background on performance, but also on the influence of performance on attainment, but we do not have clear arguments why the latter would change with tracking age.

Even after taking educational performance differences into account, children from high socioeconomic background tend to enter higher tracks than those of low socioeconomic background (*secondary effects*)^44,45^. This also applies to the Netherlands: children with the same CITO-score receive on average a higher track recommendation and attend a higher track the higher educated their parents are^46,47^. Educational decisions made at young ages depend more on family background than those made at older ages^48,49^. At a younger age, other considerations than true potential are more likely to come into play when the teachers, parents, and children choose a track^50^. This could hamper those with a disadvantaged background and compensate those with an advantaged background.

An example of hampering is that teachers would (unconsciously) underestimate children who do not talk, dress, and behave according to the cultural codes that most teachers are familiar with, which tend to be the codes of the higher classes^41^. For the same reason, they would overestimate children from the higher classes that do possess this cultural capital (a form of compensation). Of Dutch teachers, 98% indeed indicates student behavior and 43% the home situation to play a role in their recommendation, especially for students whose CITO-scores are at the border of two tracks^51^. It is difficult to assess whether these considerations are the result of teacher bias, or whether teachers are correct in seeing them as important predictors of the chances for students to be successful in a particular track^52^. Either way, because children become more autonomous with age, teachers are expected to let the home environment weigh less in their track recommendations.

Moreover, parents from diverse socioeconomic background behave differently when guiding their children in making a track choice. Relative Risk Aversion theory proposes that all parents are concerned with avoiding downward mobility for their children^53^. Whereas entering one of the lower tracks would mean downward mobility for children of high-SES background, it does not for children of low-SES background. High-SES parents would therefore be more likely than low-SES parents to interfere in the educational careers of their children. For example, it is argued and sometimes shown that high-SES parents more often than low-SES parents put pressure on teachers to give a higher track recommendation^51,52,54^. If children are younger, they depend more on the involvement of their parents. This would make it more likely for low-SES compared to high-SES children to enter a track below their true potential (hampering), and more likely for high-SES compared to low-SES children to enter a track above their true potential (compensation). Additionally, higher tracks tend to take longer and are therefore (perceived to be) riskier, which applies especially to low-SES parents who are unfamiliar with the higher tracks themselves. This uncertainty enlarges if tracking occurs at a younger age because the timeframe ahead is longer, increasing the risk of myopic decisions for those of lower socioeconomic background^55^. As students grow older, they and their parents gain more information about the student’s abilities and chances of success, and students can increasingly make their own decisions. This makes it more likely for talented students from low-SES families to choose a high track even if it is unfamiliar to their parents^56,57^.

These different arguments for secondary effects predict, similar to the primary effects, that if tracking happens at younger age, the entered secondary track level depends more on the shared environment, curbing the expression of genetic differences between children. Because the entered track level is very decisive for the secondary educational diploma that children attain, we expect the same to apply to secondary educational attainment.

*H1. The influence of genes on educational attainment is stronger and of the shared environment weaker if tracking is delayed to a later* *age.*

So far, we have implicitly assumed that early tracking increases both hampering and compensation to the same extent. But of course, it may also be the case that either of these processes is the main driver of secondary effects.

The arguments for hampering, so why disadvantaged children are tracked below their actual level, are more applicable if the educational performance of the children is higher. Teachers may find a lack of cultural capital especially problematic for low-SES children entering the highest levels of education. Furthermore, the track suitable for low-performing children is likely to be familiar to low-SES parents, but the higher the performance level of their children, the more likely it is that low-SES parents are unfamiliar with the track that is suitable. This implies that especially among the high performers, family background (shared environment) matters instead of actual potential (genes). Therefore, if early tracking mostly induces hampering, it is predominantly among the group of high-performing children that early tracking increases the importance of the shared environment and lowers that of genes.

*H2a. The moderating effect of age of tracking on genetic and shared environmental influences on educational attainment (net of performance) is stronger if the performance level of children is higher.*

The arguments for compensation, so why children with an advantageous background are tracked above their actual level, are more applicable if the performance of the children is lower^58^. The lower the performance of a high-SES child, the higher the risk of downward mobility, so the more actions high-SES parents are expected to take to prevent this. Contrary, if high-SES children have high performance, there is no need for compensatory actions because they will attain a high track anyway. Thus, in case of compensation, family background (shared environment) makes a difference especially among low performers. Therefore, if early tracking mostly amplifies compensatory actions, one would expect that early tracking increases the importance of the shared environment and lowers the importance of genes mainly among children with low performance.

*H2b. The moderating effect of age of tracking on genetic and shared environmental influences on educational attainment (net of performance) is stronger if the performance level of children is lower.*

It is not so obvious whether one would expect mostly hampering (H2a) or compensation (H2b) to be increased by immediate compared to delayed tracking in the Netherlands. It could very well be that both are affected to the same extent, in which case the impact of tracking age on genetic and environment influences would not depend on the performance level of children. Johnson et al. ^59^ show that genetic effects on educational attainment are smaller and shared environmental effects are larger in the US than in Sweden for low intelligence, but not so much for high intelligence. One could interpret these results as support for the compensation of lack of talent hypothesis (H2b) by arguing that Sweden’s school system provides more equal opportunities than the US school system (parallel to our argument that delayed tracking provides more equal opportunities than immediate tracking).

## Results

### Average genetic and environmental effects on educational attainment

To test our hypotheses, we compare twins who are immediately tracked after primary school with twins who have their definitive tracking decision delayed to a later age (*N* = 8847 twins from the Netherlands Twin Register; see the “Methods” section). Before testing our hypotheses, we present some descriptive results for all tracking types together. As can be expected based on their greater genetic relatedness, identical twins are more similar in educational attainment (*ρ*_*MZ*_ = 0.80) than fraternal twins (*ρ*_*DZ*_ = 0.48). The same is true for educational performance (*ρ*_*MZ*_ = 0.81 versus *ρ*_*DZ*_ = 0.45). These twin correlations are in line with an ACE model. When fitting this model to the data, a large part (58%) of the variance in educational attainment stems from genetic differences ($V_y^{\rm {A}} = 0.65^2 + 0.56^2 = 0.74$; total variance $V_y^{\mathrm {T}} = 1.27$; see Model 1a in Table 1). The variance that results from shared environmental factors is 22% ($V_y^{\mathrm {C}} = 0.53^2 + 0.00^2 = 0.28$) and from non-shared environmental factors 19% ($V_y^{\mathrm {E}} = 0.20^2 + 0.45^2 = 0.25$). For educational performance, 70% of the variance is due to genetic differences, 11% to shared environmental factors, and 19% to non-shared environmental factors ($V_x^{\mathrm {A}} = 7.08^2 = 50.16$; $V_x^{\mathrm {C}} = 2.74^2 = 7.51$; $V_x^{\mathrm {E}} = 3.69^2 = 13.60;$ $V_x^{\mathrm {T}} = 71.28$). Genes thus may seem to have a lower impact and the shared environment to have a larger impact on educational attainment than on educational performance, but the 95% confidence intervals overlap slightly (*A*_*x*_: 65–76% vs. *A*_*y*_: 51–66%; *C*_*x*_: 5–16% vs. *C*_*y*_: 15–29%).

**Table 1:** Bivariate Cholesky ACE-decomposition of educational performance and attainment overall and split by timing of tracking (*N* = 8847).

	Model 1a	Model 1b	
	All	Immediate	Delayed
*Intercepts*			
Performance	–0.70 (0.14)	–1.33 (0.17)	1.79 (0.22)
Attainment	2.67 (0.02)	2.61 (0.03)	2.94 (0.04)
*Performance*			
$a_{xx}$	7.08 (0.15)	7.11 (0.22)	5.40 (0.22)
$c_{xx}$	2.74 (0.36)	3.64 (0.40)	0.98 (0.66)
$e_{xx}$	3.69 (0.07)	3.81 (0.10)	3.25 (0.13)
$V_x^A$	50.16	50.50	29.16
$V_x^C$	7.51	13.23	0.96
$V_x^E$	13.60	14.48	10.59
$V_x^T$	71.28	78.22	40.71
*Attainment*			
Common			
$a_{yx}$	0.65 (0.03)	0.66 (0.04)	0.53 (0.05)
$c_{yx}$	0.53 (0.05)	0.57 (0.06)	0.04 (0.13)
$e_{yx}$	0.20 (0.02)	0.22 (0.02)	0.19 (0.03)
Unique			
$a_{yy}$	0.56 (0.03)	0.53 (0.04)	0.64 (0.04)
$c_{yy}$	0.00 (0.23)	–0.16 (0.14)	–0.13 (0.14)
$e_{yy}$	0.45 (0.01)	0.44 (0.01)	0.43 (0.02)
Common + Unique			
$V_y^A$	0.74	0.72	0.69
$V_y^C$	0.28	0.36	0.02
$V_y^E$	0.25	0.24	0.22
$V_y^T$	1.27	1.31	0.93
-2 Log-likelihood	73,003.2	72,691.1	
AIC	44,181.2	43,913.1	

Model 1b is a multigroup model (groups: both twins immediate, both twins delayed, one twin immediate—other delayed). In model 1a the coefficients are constrained to be equal for immediate and delayed tracking. Standard errors are in parentheses. Performance and Attainment are mean-centered. Control-variables *male* and *year of birth* (mean-centered) are included in all models. The labels used in the table denote *a*: Genes, *c*: Shared environment, *e*: Non-shared environment; *x*: Performance (CITO-score), *y*: Attainment (Track level); *V*: Variance (see also Fig. 4).

Note that there are no aspects of the shared environment that influence attainment but not performance: the same factors influence both. For genetic influence this is different: 57% (0.65^2^/0.74) of the genetic impact on attainment is due to genes that also influence performance, but the remaining 43% to genes that influence only attainment. This indicates that educational attainment is for a considerable part the result of genetic characteristics that are not captured by the performance test at the end of primary school.

### Impact of delayed tracking on genetic and environmental influences on educational attainment

**Fig. 1:**
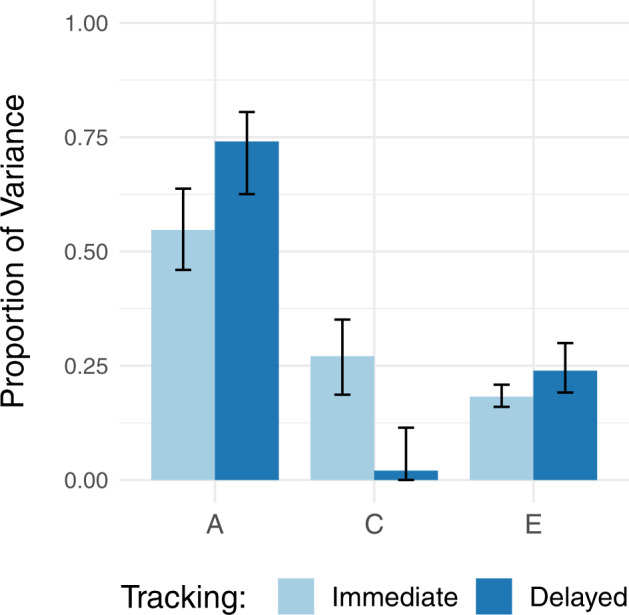
Genetic and environmental variances of educational attainment for the immediate versus delayed tracking group. Genetic (**A**), shared environmental (**C**), and non-shared environmental (**E**) variances are standardized and the error bars represent their 95% confidence intervals.

We expected that genes would be more important and the shared environment less important for educational attainment if definite tracking is delayed to a later age instead of immediately after primary school (see H1). In line with this, Model 1b and Fig. 1 show that the influence of genes on attainment is larger and of the shared environment lower when tracking is delayed. Genes account for 55% of the variance (0.72 of 1.31) in case of immediate tracking and 74% (0.69 of 0.93) in case of delayed tracking. Note that the 95% confidence intervals are just overlapping (see Fig. 1) and that the standardized effects differ not because the unstandardized genetic effects differ but because the total variance differs. The shared environment has a substantial impact (27%; 0.36) for immediately tracked children, but no significant impact when tracking occurs at a later age (2%; 0.02). We did not hypothesize on the non-shared environment and there are no significant differences to report either: 18% (0.24) for immediate versus 24% (0.22) for delayed tracking. For the group that has no information on type of tracking, the estimates are 54% for genes, 26% for shared environment, and 21% for non-shared environment (results not shown). The differences between the variance components of immediate versus delayed tracking could not be ignored statistically: Model 1a fits the data significantly worse than Model 1b (Chi^2^(22) = 312.1, *p* < 0.001, two-sided). Part of the differences between immediate and delayed tracking could be due to unmodelled gene–shared environment correlation. For immediate tracking, presence of gene–shared environment correlation would bias genetic influences downwards and shared environmental influences upwards. For the delayed tracking group such bias would be almost absent, as the shared environmental value is close to zero (see the “Methods” section).

We argued that the differences between immediate and delayed tracking may be produced both by primary and secondary effects. If delaying tracking reduces especially primary effects, one would expect to see sizable differences between immediate and delayed tracking in the variance components of educational attainment that are common to performance. Indeed, the proportion of the common variance that is due to genes, the shared environment, and non-shared environment is, respectively, 53%, 40%, and 6% for immediate tracking, while it is 88%, 0%, and 11% for delayed tracking. When looking at the unstandardized common variances, we see that the much larger shared environmental influence for immediate (0.57^2^) than delayed tracking (0.04^2^) is largely responsible for this. If delaying tracking reduces secondary effects, one would have expected the variance unique to attainment to be affected. However, the differences in the unique variance components between immediate and delayed tracking are modest: 57%, 5%, and 38% (immediate) compared to 66%, 3%, and 31% (delayed). In other words, the larger genetic and lower shared environmental effects associated with delayed tracking seem for a major part driven by a reduction in primary effects, and to a much lesser extent by a reduction in secondary effects.

### The impact of delayed tracking moderated by performance

**Fig. 2:**
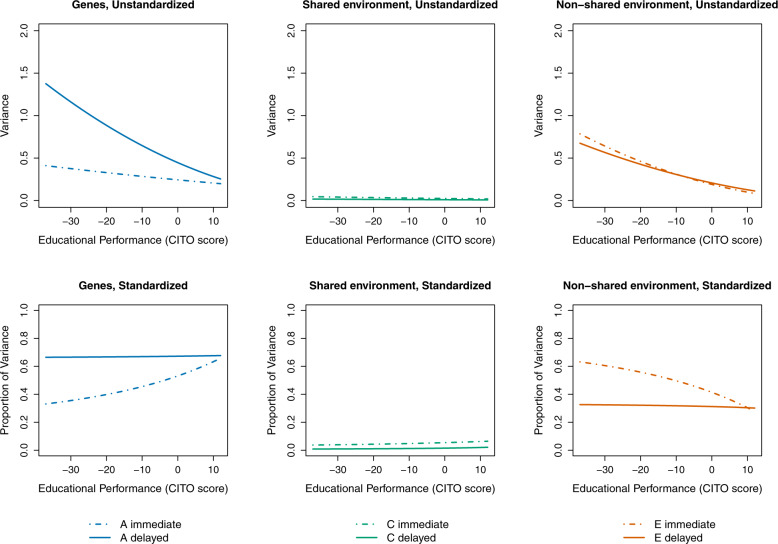
The unique genetic and environmental variances of educational attainment for immediate versus delayed tracking as moderated by educational performance. Unstandardized variances shown in the top panels and standardized variances in the bottom panels. Genetic variance shown in the left, shared environmental variance in the middle, and non-shared environmental variance in the right panels. Educational performance (CITO) scores on the x-axis are mean-centered.

However, we expected that the reduction in secondary effects by delaying tracking may not be the same for everybody: it could be that delaying tracking leads to more equal opportunities especially for high-performers (H2a) or low-performers (H2b). Model 2 in Table 2 shows how genetic and environmental influences are moderated by performance for immediate and delayed tracking. For example, the genetic effect unique to attainment is estimated to be $V_{yy}^{\mathrm {A}}|X = \left( {0.49 - 0.00 \times {\mathrm {Performance}}} \right)^2$ for immediate tracking while it is $V_{yy}^{\mathrm {A}}|X = \left( {0.67 - 0.01 \times {\mathrm {Performance}}} \right)^2$ for delayed tracking. For ease of interpretation, we plotted in Fig. 2 the results of the unique variance components of Model 2. The top graphs show unstandardized effects (raw variances) and the bottom graphs standardized effects (variances as proportion of total variance).

**Table 2:** Bivariate Cholesky ACE-decomposition of educational performance and attainment split by timing of tracking and moderated by educational performance (*N* = 8847).

	Model 2	
	Immediate	Delayed
*Intercepts*		
Performance	−1.34 (0.17)	1.77 (0.22)
Attainment	2.47 (0.03)	2.90 (0.22)
*Performance*		
$a_{xx}$	7.19 (0.22)	5.39 (0.22)
$c_{xx}$	3.50 (0.44)	1.10 (0.58)
$e_{xx}$	3.78 (0.10)	3.24 (0.13)
*Attainment*		
Common		
$a_{yx}$	0.71 (0.04)	0.56 (0.05)
$a_{yx}^\prime$	0.01 (0.00)	0.00 (0.00)
$c_{yx}$	0.62 (0.07)	0.08 (0.13)
$c_{yx}^\prime$	0.00 (0.00)	0.01 (0.01)
$e_{yx}$	0.25 (0.02)	0.19 (0.03)
$e_{yx}^\prime$	0.00 (0.00)	0.00 (0.00)
Unique		
$a_{yy}$	0.49 (0.04)	0.67 (0.05)
$a_{yy}^\prime$	−0.00 (0.00)	−0.01 (0.01)
$c_{yy}$	−0.16 (0.17)	−0.10 (0.17)
$c_{yy}^\prime$	0.00 (0.00)	0.00 (0.01)
$e_{yy}$	0.44 (0.01)	0.46 (0.03)
$e_{yy}^{\prime}$	−0.01 (0.00)	−0.01 (0.01)
−2 Log-likelihood	72,386.9	
AIC	43,644.9	

Model 2 is a multigroup model (groups: for each zygosity both twins immediate, both twins delayed, one twin immediate—other delayed). Standard errors are in parentheses. Performance and Attainment are mean-centered. Control-variables male and year of birth (mean-centered) are included in all models. The labels used in the table denote *a*: Genes, *c*: Shared environment, *e*: Non-shared environment; *x*: Performance (CITO-score), *y*: Attainment (Track level) (see also Fig. 4).

The top and bottom left graph show that the difference in unique genetic effects on attainment between immediate and delayed tracking is absent for high performers but much larger for low performers. This result is contrary to H2a and in line with H2b, which states that delayed tracking is associated with smaller secondary effects especially for students with lower performance scores. However, the same hypothesis predicted that the difference in the unique shared environmental effect between immediate and delayed tracking would also be strongest among low performers. But as we can see in the middle top and bottom graph, the effect of the shared environment for immediate compared to delayed tracking does not depend on students’ performance. The top and bottom right graph show that the non-shared environmental effects do not differ much between immediate and delayed tracking for high performers, but for low performers the non-shared environmental impact is larger for immediate than delayed tracking (which is what was predicted by H2b, but not found, for the shared environment). For the overall effects (common and unique variance components summed), the same patterns apply, but then the shared environment is for all performance levels higher for immediate compared to delayed tracking (see Supplementary Figure). Altogether this means that for the delayed tracking group, relative genetic and environmental influences do not depend on performance level, but for the immediate tracking group, only high performers can rely to a large extent on their talent when it comes to educational attainment while low performers are more dependent on non-shared environmental factors.

Model 2 reflects the patterns in the data better than Model 1b, where the impact of delaying tracking was assumed to be equal across performance levels (Chi^2^(18) = 304.2, *p* < 0.001, two-sided). Model 2 also fits the data better than a model where genetic and environmental effects are moderated by performance level, but where this moderation is constrained to be equal for immediate and delayed tracking (Model not shown; Chi^2^(6) = 13.0, *p* = 0.044, two-sided). Still, it is good to realize that the differences between immediate and delayed tracking for the low performance scores may be unreliably estimated. The reason is that only a small percentage of those with low performance scores delay tracking, as will be discussed in the next section.

### Selection into immediate versus delayed tracking

We found that delaying tracking is associated with a lower influence of the shared environment and a larger influence of genes, and that for genes this effect of delaying tracking is larger if the performance level of children is lower. However, the decision to be tracked immediately or later may itself depend on family background and performance level. In that case, the observed differences between delayed and immediate tracking may not be an effect of age of tracking but the result of a spurious association.

Table 3 shows that family background, at least when measured by parental education, does not seem to influence whether children are immediately tracked or not. No matter what the education of parents, the percentage of children with delayed tracking hoovers around the average of 27.0% (from low to high parental education: 23.5%, 27.1%, 29.3%, 25.9%). This forms reassurance that parental SES does not confound the observed association between delayed tracking and genetic and environmental influences on educational attainment. This reassurance is strengthened by the findings of others that parental SES is not related to relative genetic and environmental influences on intelligence^37^ and performance^38^ in the Netherlands.

**Table 3:** Number and percentage with immediate and delayed tracking by parental education.

	Tracking		
Parental Education	Immediate	Delayed	Total
Primary/lower secondary (basis, mulo, mavo, lts)	634	195	829
	*76.5*	*23.5*	*100.0*
Upper secondary (havo, vwo, mbo)	1466	546	2012
	*72.9*	*27.1*	*100.0*
Lower Tertiary (hbo, few years university)	1160	481	1641
	*70.7*	*29.3*	*100.0*
Upper Tertiary (university, postdoctoral)	740	258	998
	*74.1*	*25.9*	*100.0*
Total	4000	1480	5480
	*73.0*	*27.0*	*100.0*

*F*(3.00, 9148.92) = 2.00, *p* = 0.111, two-sided (Pearson Chi^2^-test with Rao–Scott second-order correction for dependence within twin pairs). Percentages are in italics.

Table 4 shows that the decision to delay tracking does depend on the performance of the child. The chance to delay tracking generally increases with performance level (except for the highest performers, which makes sense because a ceiling effect means the pre-university track can only be combined with a lower but not a higher track). Because performance is thus a potential confounder it is important to control for it in the analyses. This is effectively what happened when we included performance as a moderator in Model 2 (Table 2). The results show that genetic and shared environmental influence still differ between immediate and delayed tracking once split by performance levels (see Supplementary Figure). In other words, performance level does not seem to act as a confounder either. Of course, we cannot rule out that there are other variables that act as confounders, but it is reassuring that it is not the case for parental SES and performance.

**Table 4:** Number and percentage with immediate and delayed tracking by educational performance (CITO-score).

Performance score (corresponding track level)	Tracking		
	Immediate	Delayed	Total
500-518	153	10	163
(VMBO Basis)	*93.9*	*6.1*	*100.0*
519-525	337	25	362
(VMBO Basis/Kader)	*93.1*	*6.9*	*100.0*
526-528	287	34	321
(VMBO Kader)	*89.4*	*10.6*	*100.0*
529-532	453	107	560
(VMBO Theoretisch & Gemengd)	*80.9*	*19.1*	*100.0*
533-536	585	203	788
(VMBO Theoretisch & Gemengd/HAVO)	*74.2*	*25.8*	*100.0*
537-539	488	244	732
(HAVO)	*66.7*	*33.3*	*100.0*
540-544	894	510	1,404
(HAVO/VWO)	*63.7*	*36.3*	*100.0*
545-550	1,187	404	1,591
(VWO)	*74.6*	*25.4*	*100.0*
Total	4,384	1,537	5,921
	*74.0*	*26.0*	*100.0*

*F*(6.89, 22762.75) = 31.67, *p* < 0.001, two-sided (Pearson Chi^2^-test with Rao–Scott second-order correction for dependence within twin pairs). Percentages are in italics. The CITO-scores and their corresponding track levels are based on Ministry of Education, Culture and Science (2016)^93^, but note that norms change slightly throughout the years.

## Discussion

In various countries, children are tracked into ability groups already at an early age. There are worries that this creates unequal opportunities for children of different socioeconomic background to develop their talents. It is hard to establish whether this is truly the case, because talents and family influence are difficult to disentangle. We therefore took a behavioral genetics approach and showed that delaying tracking to a later age is associated with a larger genetic influence and a lower shared environmental influence on educational attainment of Dutch twins. This supports the idea that delayed tracking provides an opportunity structure in which children are better able to realize their genetic potential and in which their educational attainment depends less on their family background.

One reason why we expected this greater equality of opportunity with delayed tracking is because delaying tracking can make children’s school performance depend less on their family background and more on their genetic makeup, and children’s performance is central in the track they enter (i.e., reduction of primary effects of family background on educational level that work via children’s performance). Another reason has to do with the fact that children can still enter a different track level than would be expected based on their performance: delaying tracking could make it less likely that such incongruent track choices happen because of children’s family background, but rather because of children’s genetic predispositions that were not reflected in their performance (i.e., reduction of secondary effects). Our results suggest that especially the reduction of primary effects could be pivotal. We base this conclusion on the finding that the differences between immediate and delayed tracking are driven mostly by genetic and shared environmental factors that are common to both performance and educational attainment, and to a lesser extent by genetic and shared environmental influences unique to educational attainment.

These findings support transactional models of development as found in the literature on gene–environment interplay: a context that gives children more autonomy to form their direct learning environment means their learning opportunities will fit their genetic predispositions better^31^. In general, children are thought to have more autonomy at an older age. So, in case of our study, having been assessed at a later age may have given children the chance to catch up if they were held back at a younger age by their family background. Interestingly, this interpretation runs counter to findings in the British context where genetic influence on educational performance does not increase with age^60^, and to findings from a non-genetic study that institutional differences especially impact secondary and not primary effects^56^. The differences that we find are rather large for an age difference of one to three years. While unmodeled gene–environment correlation may have overstated the differences between immediate and delayed tracking somewhat, there is also a possible substantive explanation. It may not (only) be that children were assessed at a later age for the delayed tracking group, but (also) that they were assessed in secondary instead of primary school. This may have given children the chance to develop and show their capabilities within the setting that defines which qualities are required. These qualities may differ in some respects from those required in primary school, or from those captured by a performance test. We indeed showed in our analyses for all tracking types together that over 40% of the genetic influence on educational attainment in secondary school is due to genes that did not influence performance in primary school.

We further showed that genetic influence was smaller with immediate tracking for low-performing students, but for high-performing students genetic influence was high no matter the timing of tracking. This fits the idea that early tracking leads to inequality of opportunity especially because it creates possibilities for children from advantaged backgrounds to be compensated for low performance. Support for this form of secondary effects would have been even stronger if immediate tracking was associated with a larger shared environmental influence especially among low-performing students, but this pattern occurred for the non-shared environment instead. It could therefore also be that, while delayed tracking mitigates biases in track placement related to family background for students of all performance levels, in addition it corrects "random" placement errors that are likely to occur for low performers. To understand the underlying mechanisms better, a relevant next step would be to collect and include detailed measures of involved processes, such as how parents aid their children in their schoolwork and school choices^61^.

Our study complements previous studies that exploit country variability in age of tracking^4,62–64^, institutional reforms^65–67^, or a combination of both^44,68^. These studies also conclude that delaying tracking decreases inequality of opportunity but were not able to disentangle genetic and environmental influences. This does not mean our study settles the matter: our design is not perfect either. The most important limitation is that whether children delay tracking is not exogenously controlled, which the non-genetic literature deals better with by using difference-in-difference^69^ or instrumental variable approaches^26^. This leaves open the possibility that children that choose to delay tracking are somehow also the ones that would have been better in realizing their genetic potential if they would have been tracked immediately. If such selection takes place, we showed that it is unlikely that it is based on socioeconomic background or children’s performance level. Still, this does not rule out the possibility of other confounders. For example, it may be the case that a certain type of primary school is more likely to advice children to delay tracking *and* is better in providing equal opportunities. It is likely that these considerations do not apply to the entire sample: children for whom there is little choice between different high schools because they live in rural areas, for example, can be considered as part of a group in which timing of tracking is almost exogenously controlled.

Another concern that is sometimes voiced is that twins form a “special group” and are not representative of the general population. Although twins could indeed behave differently or be treated differently by parents or teachers than regular siblings, empirical studies find little proof for this^70–72^. Exciting is the development of genetically informative data and designs with the inclusion of molecular genetic data^73,74^. A Genome-Wide Association Study (GWAS) is a data-mining technique that identifies the associations between a trait, such as educational attainment, and genetic loci in DNA. Results of a GWAS may be summarized into a polygenic score, which can be seen as someone’s predisposition to the trait. If our results hold, one would expect future research to find that the effect of the polygenic score for education is larger for children who are tracked at a later age. GWASs should not be seen as the ultimate solution though^74^. A GWAS needs many participants for example, so is often based on data from numerous societies. This means that it captures only genetic effects that are nearly universal in all societies. When studying the impact of the wider context on genetic influences, it is important to capture also those genes that have an influence in certain subpopulations or are sensitive to the way the context is organized^75^. Put otherwise, in our case one would like to perform GWAS conditional on tracking age, but this will not be possible anytime soon. The way forward for studying genetic influences will therefore be to use and combine twin studies and GWASs, each with their own advantages and disadvantages, as a form of triangulation.

We have focused on inequality of opportunity, but there are other important forms of inequality, related to equity, that can be affected by tracking age. While the larger genetic influence observed with delayed tracked may be a sign of more equality of opportunity, it could also mean that those with lower potential get less adequate education than they would in case of immediate tracking. However, based on the findings of non-genetic studies, this is not likely: later tracking tends to be better for the education of low-performers^4^. In other words, delayed tracking could very well lead to equality of opportunity *and* equity.

If, despite the limitations of our study, our observed findings were to reflect a causal effect of delayed tracking, the most obvious policy advice to countries with early tracking that seek to combat inequality of opportunity would be to delay definite tracking to a later age. We showed that there is a serious risk of a mismatch between talents and track level if any assessment is set in stone already at the end of primary school. To avoid cutting students off from the best fitting educational career already at an early age, it may be more logical to assess, or at least reassess, the track level of a child at some point in secondary school.

## Methods

### Data

The Netherlands Twin Register (NTR) started in 1986 to approach parents to register their new-born twins^76^. Additionally, twins or the parents of young twins can always sign up later if they did not register when they were approached or if they were not reached, and this possibility is actively promoted by the NTR through several channels. Roughly every 2 years parents receive a survey about the twins’ behavior, health, and educational performance until the age of 12 years. From 1998 onward, the twins’ primary school teachers are asked to report on the twins at the ages 7, 9/10, and 12. Twins are approached for self-reports at the ages 14 (in the years 2005–2013), 16 (2005–2012), and 18 (2005–2007). Written informed consent was obtained from parents and the data collection was approved by the medical ethical review committee of the VU Medical Center Amsterdam (NTR25052007). These data are part of what is called the Young NTR (YNTR) and this is the part of the NTR that we need for our purposes. The recruitment efforts of the NTR resulted in an estimated coverage of 52% of all Dutch twin-pairs born between 1987 and 2017 and 29% of those born between 1970 and 1981 (other birth cohorts are less well represented). For more detailed information on the NTR see refs. ^77–79^.

### Selections and selectivity

We study twins who were born between 1986 and 1999 because 1986 is the first cohort for which our moderating variables (delayed tracking, educational performance) are available and 1999 the last cohort for which our dependent variable (secondary educational level) is available. We study those in regular education and not those in special education, which accommodates children with, e.g., disabilities. We further focus on those children that are in secondary school and not still in primary school when their parents took the survey around the children’s age of 12. For this population, there are 4706 twin pairs in the register for which both twins are observed, and 993 where only one twin met the selection criteria or was observed. We require data from parental reports (age 12), a self-report (age 14 through 18) and standardized test results at age 12, but NTR does not require participation across all surveys. This is the major reason why information on one or more of the analyzed variables may be missing. Our method can deal with missing information on the dependent variable through Full Information Maximum Likelihood. Those with missing on the grouping variable (delayed versus immediate tracking) are included as a separate group (see "Analytical Strategy"). Twins who have missing on the moderator (educational performance) are excluded, but if their co-twin does have information for performance that co-twin is still included. There are 8847 twins from 4941 pairs that meet this requirement (see Table 5 for descriptive information).

**Table 5:** Descriptive information on the variables used in the analyses for all twins together and for identical and fraternal twins separately.

	Mean	SD	Min	Max	*N*
*All twins*					
Attainment	2.76	1.11	0	4	5579
Performance	537.99	8.44	501	550	8847
Delayed tracking	0.26		0	1	5921
Year of birth	1992.46	3.33	1986	1999	8847
Male	0.46		0	1	8847
*MZ twins*					
Attainment	2.79	1.10	0	4	1983
Performance	538.15	8.45	501	550	3036
Delayed tracking	0.28		0	1	2088
Year of birth	1992.25	3.36	1986	1999	3036
Male	0.44		0	1	3036
*DZ twins*					
Attainment	2.74	1.11	0	4	2951
Performance	537.90	8.44	502	550	4776
Delayed tracking	0.25		0	1	3244
Year of birth	1992.57	3.31	1986	1999	4776
Male	0.48		0	1	4776

Monozygotic (identical) twin correlation $\rho _{{\mathrm {MZ}}} = 0.80$ for Attainment (track level) and $\rho _{{\mathrm {MZ}}} = 0.81$ for Performance (CITO-score). Dizygotic (fraternal) twin correlation is $\rho _{{\mathrm {DZ}}} = 0.48$ for Attainment (track level) and $\rho _{{\mathrm {DZ}}} = 0.45$ for Performance (CITO-score).

Participants in twin registers are known to have a relatively high socioeconomic background^80^. In our analytic sample, the problem does not seem to be very severe. About 35% of the fathers and 25% of the mothers is higher educated. In the general population, about 32% of men aged 35–55 in 2005 was higher educated and for women this was 26% (we reckoned this reference group is closest to the age ranges of the parents in our sample)^81^. There is a larger overrepresentation of children without a migration background. In our sample, 93% did not have a migration background, while for children in the general population who were aged 12 between 1998 and 2011 this varies between 78% and 80%^82^.

### Measures

We measure our dependent variable, *educational attainment at the secondary level*, with the secondary educational track level that is last reported by the student. Ideally, we would have a student’s final track level, but it differs per student at what age(s) track level is recorded. We use the surveys administered to the twins around the ages 14, 16, and 18. In case of recordings at multiple ages, we took the recording when the student was oldest because this is the track that is most likely to have been finished by a student. In the Dutch educational system, the lowest tracks (VMBO-basis, VMBO-kader, VMBO-gemengd/theoretisch; 4 years) prepare for different levels of senior secondary vocational school (MBO). The senior general secondary education track (HAVO; 5 years) prepares for tertiary vocational college (HBO). The academic track (VWO/Gymnasium; 6 years) prepares for university. Based on this, we assigned the following scores to the different possible tracks: 0 = VMBO-b; 1 = VMBO-k; 2 = VMBO-g/t; 3 = HAVO; 4 = VWO/Gymnasium. However, the surveys at ages 14, 16, and 18 combined multiple VMBO-tracks into one answer category. In surveys administered in the years 2004–2008, there was only one “VMBO” answer category. We assigned the score 1.5 to this. This is the average of the VMBO-tracks roughly weighted by the number of students enrolled in each VMBO track according to the surveys at age 12 where all VMBO-tracks were separate answer categories. In the years 2009–2014, the answer categories were “VMBO” and “VMBO-theoretisch”. We assigned 0.5 to “VMBO”, which is the average of VMBO-b and VMBO-k. This assumes that students in VMBO-gemengd would answer “VMBO-theoretisch”, which we assigned its original score (2). In some surveys, students were asked “which track level have you followed or are your currently following?”, resulting sometimes in multiple track levels mentioned. In those cases, we took the average score of the track levels mentioned as a best guess. When students already left secondary school and mentioned a follow-up vocational or tertiary educational level, we assigned the secondary track level that is required to enter that vocational or tertiary educational level. We treat the variable as continuous in our analyses, which means we assume equal distances between the educational categories although they will not be exactly equal. This simplifying assumption is quite common and Schröder and Ganzeboom^83^ show that it is not very problematic and in the Netherlands performs better than analyzing years of education.

We create a dichotomous variable that indicates whether *tracking is delayed to a later age* or not. The timing of definitive tracking differs between children. Some students enter secondary school in a class where all students have the same track level (i.e., a homogeneous class), which means that the track they will pursue is decided immediately after primary school and the decision is based on the track recommendation of their primary school teacher. Other students start secondary school in a class that combines two or more track levels (i.e., a heterogeneous class), which means that the track they will pursue is still undecided. After one or two years in this heterogeneous class (sometimes even three depending on the school), the decision is made which track the student will take. This decision is taken by the secondary school teachers and is not based on the track recommendation that the primary school teacher previously gave. We categorize a child to have been in a heterogeneous class, and thus to have experienced delayed tracking, if parents indicate that their child is in two or more track levels in the survey to the parents administered around the age of 12. We base the variable primarily on the information provided by the mother because they responded more often than fathers. But if mother’s response is missing, we use the information provided by the father.

*Educational performance* is based on a national test (CITO test) that is administered over a period of 3 days in the final year of primary school (around the age of 12) and plays an important role in the track recommendation given to students. The test consists of multiple-choice items that capture Dutch language, mathematics, and study skills. The test uses Item Response Theory to produce a final total score, and this score is converted to a standardized score such that scores are comparable over the years. Part of this procedure is that a child is compared to all other students who took the test in that year. Schools are not required to administer the CITO test, but it is estimated that 70–80% of all students participated. Moreover, participating schools did not differ from the total population of schools in terms of region, school size, degree of urbanization, and percentage of low-SES students^84^. The CITO scores in the YNTR can be reported by the parents, teachers, or the children themselves. The national average score is around 535 on a scale of 501–550, with a standard deviation between 9 and 10^84^. The average in our sample is somewhat higher (538) and the standard deviation somewhat lower (8.44). Reasons are probably that those from high-SES families and without a migration background are somewhat overrepresented in the YNTR, and higher scores may be more easily remembered and reported.

The NTR determines *zygosity* of same-sex twins, i.e., whether they are identical or fraternal twins, with a standard set of questionnaire items on physical resemblance and in subgroups based on DNA or blood group polymorphisms. The questionnaire items determine zygosity for the YNTR with an accuracy of 97.2%^77^.

We control in all models for sex by including a dummy variable *male*, and for the *year of birth* of the twins. We also performed our analyses while controlling for *age at measuring track level*, which did not lead to qualitatively different results (available upon request). We left age out in our final analyses to prevent losing cases with information on age missing (mean age = 15.80, SD = 1.54, range = 13–21, *N* = 4934).

For our descriptive results on selection into immediate or delayed tracking, we look at *parental education* defined as highest education of either parent. We use the parental report at age 10, or at age 7 if parental education is missing at age 10. We merged the original answer categories into 4 categories: 1 = Primary and Lower Secondary (Primary school, MULO, MAVO, LTS); 2 = Upper Secondary (HAVO, VWO, MBO); 3 = Lower Tertiary (HBO, few years HBO/university); 4 = Upper Tertiary (University, Postdoctoral).

### Analytical strategy

**Fig. 3:**
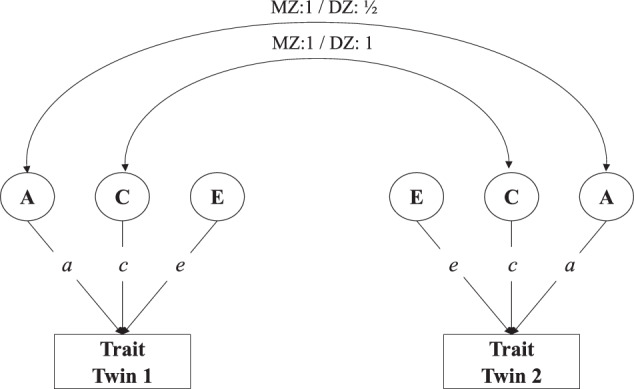
Classical twin design. The variance in a trait is decomposed into additive genetic (labeled **A**), shared environmental (**C**), and non-shared environmental influences (**E**) by using the difference in genetic relatedness between monozygotic (MZ) and dizygotic (DZ) twin pairs.

We analyze the twin data with genetic structural equation modeling to test our hypotheses in OpenMx version 2.17.3.71^85^ in R version 4.0.1^86^. Siblings tend to be similar in educational attainment and other traits because siblings share genetic material and grow up in a similar environment. To disentangle these genetic and shared environmental influences, the classical twin design compares identical (monozygotic = MZ) with fraternal (dizygotic = DZ) twin pairs because they grow up in equally similar environments but differ in genetic relatedness (100% versus ∼50%, respectively)^87^. If MZ twins are more alike than DZ twins, this is consistent with genetic influences being of importance. Data from MZ and DZ twin pairs allow decomposing the variance of a trait into additive-genetic influences (conventionally labeled: A), common or shared environmental influences (C), and individual-specific environmental influences, including measurement error (E). Figure. 3 shows the classical twin design, also called ACE-model, where the effect of the latent components A, C, and E on educational attainment are given by their path coefficients *a*, *c*, and *e*. Because the latent factors are scaled by setting their variances at 1, the total variance of educational attainment is given by the square of these path coefficients: $V = V_{\mathrm {A}} + V_{\mathrm {C}} + V_{\mathrm {E}} = a^2 + c^2 + e^2$. The covariance in educational attainment between twin 1 and twin 2 is ${\mathrm {Cov}}_{{\mathrm {MZ}}} = a^2 + c^2$ for MZ twins and ${\mathrm {Cov}}_{{\mathrm {DZ}}} = 0.5a^2 + c^2$ for DZ twins. Although we will employ more advanced versions of this classical twin design to test our hypotheses, the principle remains the same.

To test if *a* and *c* differ between children who are immediately tracked (*N* = 4384) and those with delayed tracking (*N* = 1537), we fit a multigroup structural equation model with a group for twins who are both tracked immediately, a group for twins who both have tracking delayed, and a discordant group for twins where one is immediately tracked and the other has tracking delayed. We also include those with information missing on the variable “delayed tracking” as a separate group (*N* = 2926). This way, we do not lose information of these persons or of their twin sibling that can be used to estimate other parts of the model. In our data, 4% of MZ twin pairs and 13% of the DZ pairs were in the discordant group. This approach allows checking whether constraining the path coefficients *a* and *c* for those with immediate versus delayed tracking to be equal gives a significantly worse fit than if they are allowed to differ. We look both at the raw, unstandardized, values of the variance components *a*^2^ and *c*^2^ as well as the proportion of the total variance that is explained by A and C, as both can provide valuable information. On the one hand, relative, standardized, values take into account that the total variance may differ between the immediate and delayed group even if the effects of genes and the environment do not differ (e.g., because the children in one group are more similar than in the other in terms of genetic makeup and/or (non-)shared environmental background). On the other hand, the standardized values obscure whether a component differs because its own raw value differs or that of another component (or both).

Because research on inequality of opportunity usually does not formulate expectations on E, we have no hypothesis on E either. Still, there may be differences in E between the immediate and delayed groups. On the one hand, to the extent that children’s own choices are not genetically steered, more autonomy would mean that shared environmental effects give way to non-shared environmental effects (instead of the theoretically predicted genetic effects) and thus that E becomes larger with delayed tracking. On the other hand, even without systematic bias in track placement related to family background, children will not always enter the track that fits their potential. Such "random" errors in track placement are more likely to be corrected, and thus E to be lower, when tracking is delayed than when it is immediate. Therefore, we allow the influence of E to differ.

**Fig. 4:**
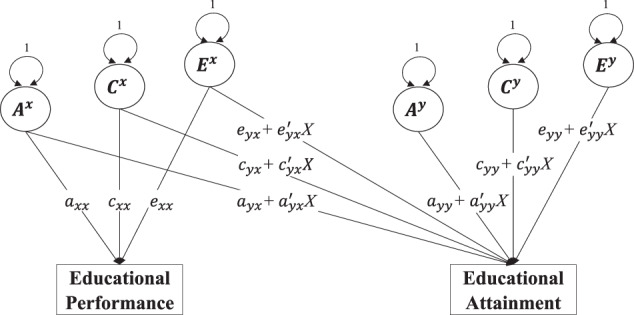
Bivariate twin moderation model with Cholesky decomposition: effects on attainment moderated by performance. The genetic (**A**), shared environmental (**C**), and non-shared environmental (**E**) effects on educational attainment are divided into a part that is common with performance (see paths *a*_*yx*_, *c*_yx_, and *e*_*yx*_) and a part that is unique to attainment (see paths *a*_*yy*_, *c*_*yy*_, and *e*_*yy*_). The size of these paths depends on the moderator educational performance (*X*). For simplicity, only one twin is shown, but the second twin could be added as in Fig. 3.

We also hypothesized that the difference in the effects of A and C between the immediate and delayed group may depend on the performance level of children. We test this by including performance level as a continuous moderator in the model and allowing its effect to differ between both the immediate and delayed group. Because performance level is genetically influenced itself and not a pure environmental variable, it cannot simply be included as a continuous moderator in the model. As a solution, we can use a bivariate model where not only the variance of educational attainment but also that of performance is decomposed into genetic and non-genetic variance components (see Fig. 4)^88,89^. The genetic and environmental factors that influence educational performance may be (partly) the same as the ones that influence educational attainment, but there may also be genetic and environmental factors that influence only educational attainment and not performance. A so-called Cholesky decomposition model reflects this by dividing the genetic and environmental effects on educational attainment into a part that is common with performance (see paths *a*_*yx*_, *c*_*yx*_, and *e*_*yx*_) and a part that is unique to attainment (see paths *a*_*yy*_, *c*_*yy*_, and *e*_*yy*_). Moreover, the size of these paths can be allowed to depend on performance level. For example, *a*_*yx*_ becomes $a_{yx} + a_{yx}^\prime X$, where *X* is the performance level. The total variance in educational attainment is then conditional on *X* and given by $V_y|X = V_{yx}|X + V_{yy}|X = ( {a_{yx} + a_{yx}^\prime X} )^2 + ( {c_{yx} + c_{yx}^\prime X} )^2 + ( {e_{yx} + e_{yx}^\prime X} )^2 + ( {a_{yy} + a_{yy}^\prime X} )^2 + ( {c_{yy} + c_{yy}^\prime X} )^2 + ( {e_{yy} + e_{yy}^\prime X} )^2$.

In this model, *c*_*yy*_ (or $c_{yy} + c_{yy}^\prime X$ when moderated) captures what were labeled secondary effects in the “Introduction” section, but the interpretation of *c*_*yx*_ is less clear-cut in this respect. It captures situations where shared environmental factors influence performance and performance in turn influences attainment (i.e., primary effects). But it also captures shared environmental factors that influence attainment directly (i.e., secondary effects), but where the same shared environmental factors happen to influence performance as well.

### Assumptions of the fitted twin models

An assumption of the twin model is that the variances in performance and attainment do not differ by the order in which the twins are born, their zygosity, and their sex, after correcting for their fixed effects. We checked this assumption by estimating a model where all variances and means were allowed to differ and then constraining the variances step by step. The results show that the assumption was not violated (see Supplementary Tables 1 and 2). Because the means of performance and attainment differed significantly by sex, with males scoring slightly higher than females, we included sex as a fixed effect in all of our main analyses.

The twin models that we fit make several other (strong) assumptions^90^. For example, one is that, for all environmental influences that have an impact on the dependent variable, identical twins share their environment on average to the same extent as fraternal twins. A second is that the effects of genetic variants at the same locus or at different loci are only additive and do not interact with one another, i.e., that there are no dominant or epistatic genetic effects. Note however, that we first examine this assumption by comparing MZ and DZ correlations: strong non-additive genetic effects would lead to substantially lower DZ (but not MZ) correlations. A third is that there is no assortative mating. If we find a substantial estimate for the influence of C, we recognize this may be caused by assortative mating. Fourth, the models assume that there is no correlation between peoples’ genes and the type of environment they grow up in.

Violations of these assumptions lead to biased estimates of the additive genetic and shared environmental effects, but it is difficult to predict in what direction because the violations work in opposing directions^90,91^. Violation of the equal environment assumption and the assumption that genetic effects are only additive lead to an overestimation of additive genetic effects and underestimation of shared environmental effects. On the other hand, if there is genetic assortative mating or gene–shared environment correlation, the models that we fit underestimate additive genetic effects and overestimate the shared environmental effects.

However, it is important to note that in this paper, we are interested in comparing the value of genetic and shared environmental effects in one context relative to that of another. To distort the tests of our hypotheses, the estimates should thus be more biased in one context than the other. Although this is possible, we have not been able to come up with evident arguments why and how the assumptions of our models would be violated differently for those tracked immediately compared to those tracked at a later age, with possibly the exception of passive gene–environment correlation. It is not very likely that the passive gene–shared environment correlation itself differs because that would require parental genes to influence their children’s shared environment differently for children with immediate versus delayed tracking. Nevertheless, the amount of bias due to ignoring passive gene–environment correlation also depends on the values of genetic and shared environmental influence, and our results showed these to differ. Verhulst and Hatemi^92^ show with simulations that the bias is smaller when either the genetic or shared environmental influence approaches zero, and obviously when gene–environment correlation itself is lower. Given the values that we found for genetic and shared environmental influence, the simulations imply that bias is practically absent for the delayed tracking group and at most modest for the immediate tracking group, which could have overestimated the differences between immediate and delayed tracking. Note finally that gene–environment correlation only distorts the estimated differences between immediate and delayed tracking if there are *real* differences between the two groups.

### Reporting summary

Further information on research design is available in the Nature Research Reporting Summary linked to this article.

## Supplementary information

Supplementary Information

Reporting Summary

## Data Availability

Netherland Twin Register (NTR) is an ongoing longitudinal study. Data may be accessed, upon reasonable request and after approval of the data access committee. Please contact the NTR (ntr.fgb@vu.nl).

## Code availability

Code for analyses and figures is available in a GitHub repository at https://github.com/antonieknigge/Code_DelayedTracking.
