# Supplementary material for: Delayed tracking and inequality of opportunity: Gene-environment interactions in educational attainment
Source: NPJ Sci Learn. 2022 May 4;7:6. doi: 10.1038/s41539-022-00122-1 (PMC9068802; doi:10.1038/s41539-022-00122-1)
Supplement: Supplementary file 1 — Supplementary Information [file 41539_2022_122_MOESM1_ESM.pdf]

Supplementary Information

Delayed tracking and inequality of opportunity: Gene-environment interactions in educational attainment

Antonie Knigge*, Ineke Maas, Kim Stienstra, Eveline de Zeeuw, Dorret I. Boomsma

**Corresponding author details*
Antonie Knigge
Department of Sociology/ICS, Utrecht University
Padualaan 14, 3584CH, Utrecht, The Netherlands;
+31 (0)30 2534541; a.knigge@uu.nl.

This PDF file includes:

Supplementary Figure
Supplementary Tables 1-2

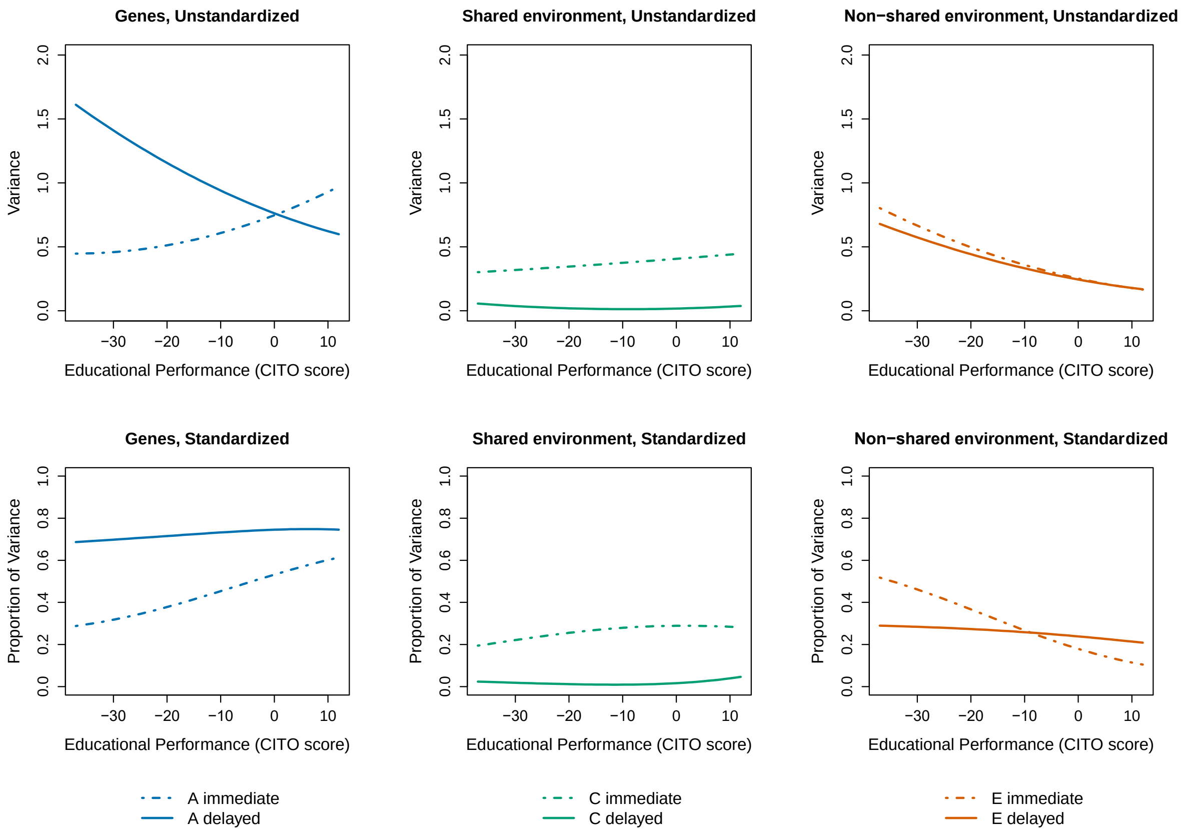

Supplementary Figure: The overall genetic and environmental variances of educational attainment for immediate versus delayed tracking as moderated by educational performance. For delayed tracking, the estimates for the path of the shared environment common with educational performance ($c_{yx} + c'_{yx}X = 0.08 + 0.01 \times Performance$) are such that they become slightly negative when educational performance scores are below around –11. Because this path is squared to obtain the variance, the variance becomes positive where it would be more appropriate if it was estimated to be negative. Negative variance estimates can be an indication that the underlying model is incorrect, for example because existing dominant genetic effects are ignored, leading to underestimation of shared environmental effects (see section "Assumptions of twin models" in main text). But negative variance estimates can also simply result from sampling variability if the true population value is close to 0. We ran a model where combinations of parameters such as $c_{yx} + c'_{yx}X$ are constrained to be positive for all values of educational performance. This model hardly changed the results because the shared environmental effects of delayed tracking are in all models very close to 0 anyway (results available upon request). It is usually not recommended to constrain parameters to be non-negative, so we use the unconstrained model in our main analyses.

Supplementary Table 1. Checking assumptions of equal variance of educational attainment for twin order, zygosity, and sex.

Model	Estimated parameters	-2 Log-likelihood	Degrees of freedom	χ^2	p
Saturated	30	15619.4	5549		
+ Equal variances twin order	24	15623.8	5555	4.31	.635
+ Equal variances zygosity	21	15623.9	5558	0.15	.992
+ Equal variances sex	19	15624.2	5560	0.63	.810

Supplementary Table 2. Checking assumptions of equal variance of educational performance for twin order, zygosity, and sex.

Model	Estimated parameters	-2 Log-likelihood	Degrees of freedom	χ^2	p
Saturated	30	60646.2	8817		
+ Equal variances twin order	24	60650.6	8823	4.38	.631
+ Equal variances zygosity	21	60653.3	8826	2.74	.434
+ Equal variances sex	19	60657.9	8828	4.54	.103
