# Supplementary material for: Delayed tracking and inequality of opportunity: Gene-environment interactions in educational attainment
Source: NPJ Sci Learn. 2022 May 4;7:6. doi: 10.1038/s41539-022-00122-1 (PMC9068802; doi:10.1038/s41539-022-00122-1)
Supplement: Supplementary file 2 — Reporting Summary [file 41539_2022_122_MOESM2_ESM.pdf]

nature portfolio

Corresponding author(s): Antonie Knigge

Last updated by author(s): Mar 15, 2022

Reporting Summary

Nature Portfolio wishes to improve the reproducibility of the work that we publish. This form provides structure for consistency and transparency in reporting. For further information on Nature Portfolio policies, see our Editorial Policies and the Editorial Policy Checklist.

Statistics

For all statistical analyses, confirm that the following items are present in the figure legend, table legend, main text, or Methods section.

n/a	Confirmed	
☐	☒	The exact sample size (*n*) for each experimental group/condition, given as a discrete number and unit of measurement
☐	☒	A statement on whether measurements were taken from distinct samples or whether the same sample was measured repeatedly
☐	☒	The statistical test(s) used AND whether they are one- or two-sided<br>*Only common tests should be described solely by name; describe more complex techniques in the Methods section.*
☐	☒	A description of all covariates tested
☐	☒	A description of any assumptions or corrections, such as tests of normality and adjustment for multiple comparisons
☐	☒	A full description of the statistical parameters including central tendency (e.g. means) or other basic estimates (e.g. regression coefficient) AND variation (e.g. standard deviation) or associated estimates of uncertainty (e.g. confidence intervals)
☐	☒	For null hypothesis testing, the test statistic (e.g. *F*, *t*, *r*) with confidence intervals, effect sizes, degrees of freedom and *P* value noted<br>*Give P values as exact values whenever suitable.*
☒	☐	For Bayesian analysis, information on the choice of priors and Markov chain Monte Carlo settings
☐	☒	For hierarchical and complex designs, identification of the appropriate level for tests and full reporting of outcomes
☒	☐	Estimates of effect sizes (e.g. Cohen's *d*, Pearson's *r*), indicating how they were calculated

Our web collection on statistics for biologists contains articles on many of the points above.

Software and code

Policy information about availability of computer code

Data collection	No software was used for data collection in this study.
Data analysis	Code for analyses and figures is available in a GitHub repository at https://github.com/antonieknigge/Code_DelayedTracking.

For manuscripts utilizing custom algorithms or software that are central to the research but not yet described in published literature, software must be made available to editors and reviewers. We strongly encourage code deposition in a community repository (e.g. GitHub). See the Nature Portfolio guidelines for submitting code & software for further information.

Data

Policy information about availability of data

All manuscripts must include a data availability statement. This statement should provide the following information, where applicable:

- Accession codes, unique identifiers, or web links for publicly available datasets
- A description of any restrictions on data availability
- For clinical datasets or third party data, please ensure that the statement adheres to our policy

Netherland Twin Register (NTR) is an ongoing longitudinal study. Data may be accessed, upon reasonable request and after approval of the data access committee. Please contact the NTR (ntr.fgb@vu.nl).

Field-specific reporting

Please select the one below that is the best fit for your research. If you are not sure, read the appropriate sections before making your selection.

☐ Life sciences ☒ Behavioural & social sciences ☐ Ecological, evolutionary & environmental sciences

For a reference copy of the document with all sections, see nature.com/documents/nr-reporting-summary-flat.pdf

Behavioural & social sciences study design

All studies must disclose on these points even when the disclosure is negative.

Study description	A classical twin study using quantitative data on educational performance and educational attainment.
Research sample	The Netherlands Twin Register (NTR) is a population-based register that recruits multiples and their family members for longitudinal research. This study included 8,847 children from birth cohorts 1986-1999. Their educational performance was assessed at age 12 and their attainment between age 14 and 18.
Sampling strategy	The NTR is a population-based register that ascertains participants based on their multiple birth status (twins, triplets, etc) or because they are related to multiple-birth individuals. They are no exclusion criteria for registration, though specific research projects may apply such criteria, as described in the manuscript. No a-priory power analysis was performed, as the study included all available data.
Data collection	The data were collected using pen-and-paper surveys and for more recent cohorts online surveys.
Timing	The NTR is an ongoing longitudinal data collection.
Data exclusions	Data excluded from twins:<br>- in special needs education<br>- that are still in primary school at age 12<br>- with missing zygosity status<br>- with missing information on educational performance (if co-twin has information on performance that co-twin is still included).
Non-participation	Information on response rates of the different NTR surveys can be found in van Beijsterveldt et al. (2013) in Twin Research and Human Genetics.
Randomization	Participants were not allocated to experimental groups.

Reporting for specific materials, systems and methods

We require information from authors about some types of materials, experimental systems and methods used in many studies. Here, indicate whether each material, system or method listed is relevant to your study. If you are not sure if a list item applies to your research, read the appropriate section before selecting a response.

Materials & experimental systems

n/a	Involved in the study	
☒	☐	Antibodies
☒	☐	Eukaryotic cell lines
☒	☐	Palaeontology and archaeology
☒	☐	Animals and other organisms
☐	☒	Human research participants
☒	☐	Clinical data
☒	☐	Dual use research of concern

Methods

n/a	Involved in the study	
☒	☐	ChIP-seq
☒	☐	Flow cytometry
☒	☐	MRI-based neuroimaging

Human research participants

Policy information about studies involving human research participants

Population characteristics	See above.
Recruitment	The majority of Twins participating in the YNTR are registered shortly after birth by their parents, and are recruited with the help of a commercial baby organization that visits parents of newborns and the 'Dutch association for parents of multiples'. Non-response analysis revealed that parents from a lower SES were less likely to return surveys, but, while statistically significant, differences were small (van Beijsterveldt et al., 2013).

Ethics oversight	The data collection was approved by the medical ethical review committee of the VU Medical Center Amsterdam (NTR25052007)

Note that full information on the approval of the study protocol must also be provided in the manuscript.
